# How lifestyle counselling supports behaviour change among Thai adults with non-communicable diseases: a rapid realist review

**DOI:** 10.1136/bmjopen-2025-115071

**Published:** 2026-09-09

**Authors:** Bonan Chen, Chaisiri Angkurawaranon, Iliatha Papachristou Nadal

**Affiliations:** 1 Institution of Psychiatry, Psychology and Neuroscience, King’s College London, London, UK; 2 Department of Family Medicine, Faculty of Medicine, Chiang Mai University, Chiang Mai, Thailand; 3 Division of Care in Long Term Conditions, King’s College London, London, UK

**Keywords:** Health Literacy, Review, PUBLIC HEALTH, Psychosocial Intervention, Behavior, Cardiovascular Disease

## Abstract

**Abstract:**

**Objective:**

Non-communicable diseases (NCDs) are major contributors to morbidity and mortality in Thailand, yet the effectiveness of lifestyle counselling within routine practice is underexplored. This rapid realist review examined how, for whom and under what circumstances lifestyle counselling supports behaviour change among Thai adults.

**Design:**

Rapid realist review following guidance from the Realist and Meta-narrative Evidence Synthesis: Evolving Standards (RAMESES).

**Setting:**

Lifestyle counselling and health-coaching interventions for NCD prevention and management delivered in Thai primary care, community settings or digitally supported programmes.

**Data sources:**

Six international and Thai databases (Scopus, Google Scholar, ProQuest, PubMed, EMBASE (Ovid) and ThaiJo) were searched for studies published between 2005 and 2025.

**Eligibility criteria:**

Empirical studies involving adults (≥18 years) in Thailand that described lifestyle counselling or coaching interventions for NCD-related prevention or management and reported outcomes.

**Data extraction and synthesis:**

Data were extracted to identify contexts (C), mechanisms (M), outcomes (O) and equity considerations. These were synthesised into context–mechanism–outcome configurations (CMOCs) and helped to form programme theories.

**Results:**

16 studies were included. 19 explanatory configurations were identified across six mechanisms: self-efficacy, social support, motivation, accountability, emotional resilience and relevance and engagement. These mechanisms and programme theories (PTs) were supported by family-centred education, routine self-monitoring with feedback, culturally or literacy-tailored materials and brief stress-regulation strategies. Barriers included low health and digital literacy, conflicting norms, short programme duration and rural workforce constraints. Facilitators included plain-language materials, low-tech or hybrid follow-up, co-designed dietary strategies and task-sharing with village health volunteers and family members.

**Conclusions:**

This rapid realist review identified six PTs, suggesting that the operation of lifestyle counselling interventions for non-communicable diseases in Thailand may be influenced by cultural norms, family and community support, village health volunteers and health service capacity. The findings highlight the potential importance of sustained follow-up, cultural and literacy tailoring and accessible modes of delivery in supporting behaviour change and self-management across different contexts. These PTs provide insight into how lifestyle counselling may operate in Thailand and may inform future research and the refinement of lifestyle counselling interventions.

**STRENGTHS AND LIMITATIONS OF THIS STUDY**

- This study applied a rapid realist review approach to examine lifestyle counselling interventions for non-communicable diseases in Thailand, enabling an understanding of how, why, for whom and under what circumstances these interventions are effective.
- The review followed the Realist and Meta-narrative Evidence Synthesis: Evolving Standards (RAMESES) guidance, enhancing transparency, consistency and rigour in the synthesis process.
- A broad search across international and Thai databases enhanced the inclusiveness of the evidence base, although a limited number of Thai-language studies were included.
- Variability in intervention descriptions, study designs and reporting limited the detail available for developing and refining explanatory theories.
- Stakeholder engagement with Thai healthcare professionals provided additional perspectives on the contextual relevance and feasibility of the programme theories; however, patients and members of the public were not involved, and the small stakeholder group may limit the perspectives represented.

## Introduction

 Non-communicable disease (NCD) is a major global health challenge, responsible for around 74% of global mortality. The majority of these occur in low- and middle-income countries.^1^ Thailand reflects this trend, with cardiovascular disease, diabetes, chronic respiratory disease and cancer now forming the leading causes of death. Based on this pattern, WHO estimates indicate that NCDs caused roughly two-thirds of deaths in 2021 and about three-quarters in recent country reporting.^1 2^ Thailand’s national policy, ‘Every Thai Family Has Three Doctors’, supports the prevention of NCDs and health promotion under universal health coverage. The policy links family caregivers, primary care teams and approximately one million village health volunteers (VHVs), enabling nationwide coverage, including rural areas.^3 4^

Many NCD risks are behaviourally mediated, with key modifiable factors including unhealthy diet, physical inactivity, poor stress management and substance use.^5–7^ Thai cultural norms and socioeconomic disparities are shaped mainly by these lifestyle factors.^8 9^ Accordingly, lifestyle counselling in Thailand promotes goal setting, motivational interviewing, health coaching and group education in both primary care and community programmes.^10^ Recent evidence from Thailand supports the feasibility of community-based lifestyle counselling through nurse-led and village health volunteer-assisted Diabetes Self Management Education and Support (DSMES) programmes within primary care settings.^11^ Relevant studies found improvements in diet, activity, medication adherence, tobacco and alcohol use and mental health by using these interventions. However, most evaluations emphasise outcomes or delivery and give limited attention to how context and mechanisms sustain behaviour change.^12–18^

Mechanisms such as self-efficacy, self-management and social support are often examined in cross-sectional surveys as predictors. However, they are rarely tested within specific interventions or theorised during implementation in Thai communities.^18 19^ As a result, evidence is fragmented; and the synthesis is limited on how contextual factors influence the adoption, effectiveness and success or failure of counselling strategies.^20 21^ Challenges in the implementation of these interventions also remain: service quality is uneven; health literacy levels and resources can be limited; and cultural norms influence behaviour change. Although VHVs are vital to counselling and follow-up, their work differs by locality. Existing research tends to consider these barriers in isolation by policy level or provider viewpoint, leaving limited synthesis of how contextual factors influence the success or failure of specific counselling strategies.^20–25^

### Initial programme theories

Drawing on behavioural and implementation literature, this review initially theorised that lifestyle counselling supports behaviour change through mechanisms including self-efficacy, motivation, autonomy, social support, emotional resilience and accountability.^26–28^ These mechanisms were selected because previous research suggests that sustainable self-management of NCDs often depends not only on knowledge provision but also on individuals’ confidence in their ability to change behaviour, internal motivation, perceived support and capacity to maintain behavioural changes over time.^17^ These initial programme theories (PTs) were informed by Social Cognitive Theory, particularly the role of self-efficacy in behaviour adoption and maintenance^29^; Self-Determination Theory regarding the intrinsic motivation and autonomy^30^ and the Capability, Opportunity, and Motivation-Behaviour (COM-B) model, which conceptualises accountability and behaviour change as the interaction between capability, opportunity and motivation.^13^

The review further proposed that these mechanisms would operate differently according to contextual conditions within Thai healthcare and community settings. Relevant contextual influences included family involvement, health literacy, socioeconomic conditions, digital access, cultural norms and the availability and engagement of VHVs.^3–10^ In Thailand, health behaviours are often shaped by family relationships, community structures, local beliefs and access to healthcare resources. It was therefore hypothesised that when counselling interventions align with participants’ social, cultural and healthcare contexts, these mechanisms are more likely to support engagement, self-management and sustained behaviour change.

### Review objective and questions

Accordingly, the main gaps include limited focus on mechanisms and context within Thai community and primary-care programmes, inconsistent evidence across contexts and under-reporting of equity considerations (eg, digital literacy, rural–urban and religious differences). This review aims to address these gaps and explain how, when and for whom lifestyle counselling for NCDs works in Thailand, by adopting a rapid realist review (RRR). While developing context–mechanism–outcome configurations (CMOCs) for PTs, it explores:

1. What contextual factors influence the adoption and effectiveness of these interventions?
2. Which mechanisms drive behaviour change and sustained self-management?
3. What outcomes are commonly reported?
4. What are the barriers and facilitators to effective implementation?

This review extends existing work by developing CMOCs and PTs specific to Thai community and primary care settings, addressing gaps in mechanism-focused explanations, equity considerations and contextual analysis.

## Methods

A rapid realist review (RRR) was conducted to synthesise evidence on lifestyle counselling interventions for NCDs in Thailand.^31 32^ RRR links context to outcomes, which makes it suitable for time-sensitive, real-world policymaking.^32^ Thus, it fits Thailand’s context, where cultural norms, socioeconomic conditions and service delivery interact to shape counselling outcomes. The review was conducted in accordance with the Realist and Meta-narrative Evidence Synthesis: Evolving Standards (RAMESES) publication guidance for realist syntheses to ensure transparency, consistency and methodological rigour^33 34^ (see online supplemental table 1 for details). This realist review also serves as a preliminary step to inform subsequent qualitative research through in-depth interviews with expert panels. It helps to develop PTs and ensure that future intervention design is evidence- and context-informed.

### Search strategy

Literature searches were conducted between 16 and 19 January 2025 and updated in June 2025 in Scopus, Google Scholar, ProQuest, PubMed, EMBASE (Ovid) and the ThaiJO database. The search covered the past 20 years (2005–2025) to capture more recent evidence on lifestyle counselling interventions for NCDs in Thailand. All study designs and publication types were included before full screening. Search terms combined keywords and subject headings, including lifestyle counselling interventions, health coaching, NCDs, Thailand and adults (online supplemental table S2) in online supplemental appendix. Filters were applied to restrict results to the specified time frame. Records published in English and Thai were considered. Thai-language studies identified during the original search process were also screened against the predefined inclusion criteria. Screening and assessment of these studies were undertaken with support from the Thai co-author (CA), who reviewed eligibility and contextual relevance.

### Selection and appraisal of paper

All search results were imported into Zotero (reference manager) for de-duplication, screening and appraisal process. Title and abstract screening were initially conducted by reviewers (BC) and (IPN) using the pre-defined inclusion and exclusion criteria (table 1 below). Obviously irrelevant articles were excluded at this stage. When the eligibility was unclear for some references, these records were retained for independent assessment by the second reviewer (IPN). IPN screened all records marked as eligible and potentially uncertain to minimise the risk of missed or incorrectly included studies.

**Table 1:** Screening inclusion and exclusion criteria

	Inclusion criteria	Exclusion criteria
Population	Studies included young, middle-aged or older adults aged 18 years or above with non-communicable diseases (NCDs) in Thailand.	Studies focused exclusively on children or adolescents aged under 18 years.
Intervention	Studies evaluated behavioural or lifestyle-related interventions for NCD prevention or management in Thailand, including lifestyle counselling, health coaching, behavioural modification, self-management support, motivational interviewing or health-promotion interventions with behavioural components.	Studies did not involve lifestyle counselling or behavioural interventions; studies focused solely on pharmacological, biomedical or clinical treatment without behavioural or counselling components.
Relevance to realist synthesis	Studies provided contextual, mechanistic or outcome-related information relevant to the development, testing or refinement of programme theories (PTs) and context–mechanism–outcome configurations (CMOCs).	Studies lacked sufficient contextual, explanatory or outcome-related details to contribute meaningfully to CMOC or programme theory development.
Methodological rigour	Studies with sufficiently clear and credible descriptions of methodology, intervention processes, data collection, analysis procedures and findings to support realist synthesis.	Studies with insufficient methodological details, unclear intervention processes, missing or inconsistent findings, unsupported conclusions or inadequate evidence linking findings to outcomes.
Articles retrieved	Published full-text peer-reviewed articles and accessible full reports.	Conference abstracts, protocols only, doctoral theses, editorials, commentaries or articles without accessible full text.
Study designs	Empirical qualitative, quantitative or mixed-methods studies, pilot studies and relevant grey literature (eg, government or organisational reports).	Narrative reviews, systematic reviews, opinion papers and single-patient case studies without broader explanatory relevance.
Language	Records published in English or Thai.	Any record not published in English or Thai.
Dates	Records published between Jan 2005 and Jan 2025.	Any record published before Jan 2005.

Full texts were then assessed independently by both reviewers (BC and IPN) for appraisal. Each study was judged for relevance and rigour. Relevance was that the study described a lifestyle- or health-related counselling or coaching intervention targeting NCDs in Thailand and reported outcomes; its contribution to developing or testing preliminary PTs and CMOCs was also considered. Rigour was judged by the integrity and consistency of the study design, data collection, and reported empirical findings. Relevant Thai language studies were assessed using the same relevance and rigour procedures as English language studies and incorporated into programme theory development where appropriate.

Studies were considered to provide unreliable evidence where there were substantial concerns regarding methodological integrity, transparency, or the credibility of findings. This included studies with insufficient description of methods or intervention processes, unclear or inconsistent reporting of data collection or analysis procedures, unsupported conclusions or inadequate evidence linking findings to outcomes. In line with realist review principles, studies were not excluded solely based on study design hierarchy. Instead, the appraisal focused on whether the data were sufficiently trustworthy and relevant to contribute to programme theory development and refinement. Studies judged to have limited rigour, but potentially valuable contextual insights were retained and independently reviewed by the second reviewer (IPN) during the synthesis process. Disagreements at any stage of screening or appraisal were resolved through discussion between the two reviewers.

### Data extraction, analysis, and synthesis

A preliminary data extraction framework was developed in Excel to record key study characteristics. These included bibliographic details, participant characteristics, intervention activities, outcomes and notes on barriers and facilitators. At this stage, relevance and methodological rigour were reassessed. Studies with insufficient or unreliable evidence were eventually excluded. Detailed data extraction was then completed in Word, where information was organised for realist analysis. The process began by identifying potential contexts (C), mechanisms (M), outcomes (O) and Equality, Diversity and Inclusion (EDI) within each study. Contexts referred to the conditions or settings influencing how the intervention operated; mechanisms were the processes or responses triggered in participants that explained why change happened; outcomes were the intended or unintended results; and EDI means ensuring fair access and valuing diverse backgrounds of involved participants. Individual CMOCs were developed from these data, with each describing a specific causal pathway reported in a study to capture variations in how mechanisms worked in different contexts.

Once CMOCs were drafted, they were compared across studies to identify patterns, similarities, contradictions and contextual variation. Initial PTs were iteratively tested and refined against the included evidence throughout the synthesis process. While contradictory findings emerged, studies were revisited to examine how differences in contexts, participant characteristics, implementation processes and regions or healthcare settings may explain the variation in the outcomes. This process allowed related CMOCs to be compared, modified, merged or refined into broader explanatory PTs: overarching explanations that link multiple CMOCs into a coherent account of what the programme does, and how and why those actions are expected to lead to different outcomes across contexts.^32^

One reviewer BC drafted the initial CMOCs and emerging PTs, while the second reviewer (IPN) independently examined approximately half of the included studies to assess the consistency and plausibility of the developing explanations. Feedback from IPN informed revisions of the CMOCs and PTs. Both reviewers subsequently met online to resolve differences, align reasoning, and refine the CMOC wording and interpretation. The final CMOCs and PTs were agreed collaboratively. Insights from the two invited doctoral students were also incorporated into the synthesis, ensuring that the final CMOCs and PTs reflected lived experience and published evidence.

As this work was initially conducted as part of a dissertation project, an additional Thai colleague (CA) with experience in NCD-related healthcare and familiarity within the Thai cultural context was later invited to review the manuscript as a third reviewer, prior to journal submission. This external review aimed to strengthen the credibility, cultural appropriateness, and interpretive integrity of the synthesis. Feedback from CA informed further refinement of the CMOCs, programme theories, and interpretation of contextual factors relevant to lifestyle and health coaching interventions in Thailand.

### Stakeholder engagement

Two Thai doctoral students based in Chiang Mai (Northern Thailand) with professional backgrounds as diabetes and NCD nurses working within the Thai healthcare system provided stakeholder input. Both had direct experience supporting adults with NCDs and familiarity with community and primary care delivery in Thailand. They reviewed preliminary programme theories for cultural and contextual fit. They commented on relevance, surprising findings, regional variation and additional literature. Their feedback informed refinement of CMOCs and programme theories. Their role was intended to provide contextual and implementation expertise rather than represent the wider population of adults living with NCDs. Participation was voluntary, anonymised and compensated with gift vouchers. As this was a literature review with no collection of personal or sensitive data, ethical approval was not required.

### Patient and public involvement

No patients and members of the public were involved in this rapid realist review.

## Results

**Figure 1:**
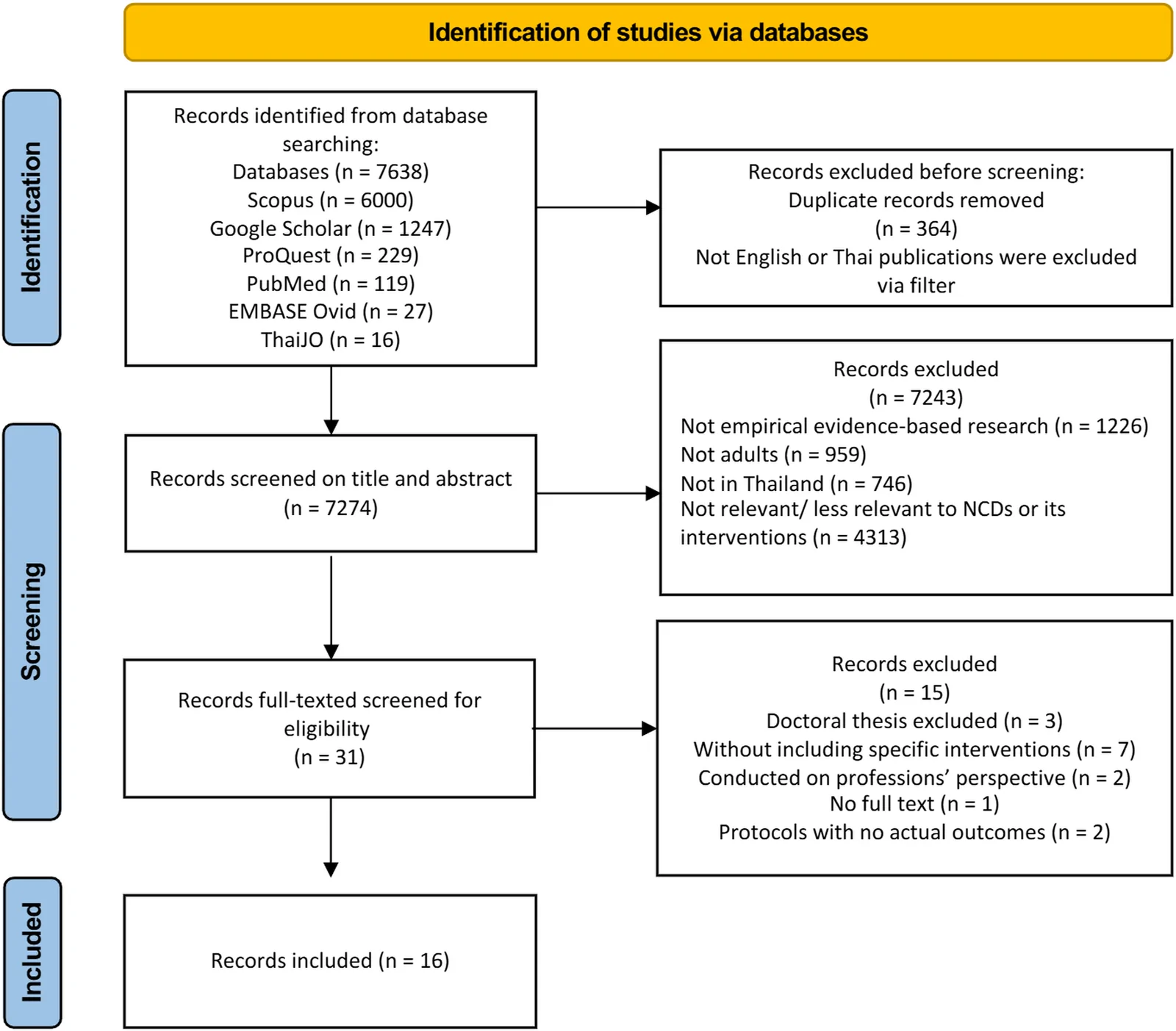
Flowchart of paper selection. NCDs, non-communicable diseases.

The Preferred Reporting Items for Systematic Reviews and Meta-Analyses (PRISMA) diagram illustrates the rapid realist review process (see figure 1). From 7638 records identified, 364 duplicates and non-English publications were removed. Of the 7274 records screened, 7243 were excluded for not meeting inclusion criteria. 31 full texts were assessed and 15 were excluded (no specific intervention, doctoral theses or no outcome data). Finally, 16 studies were included.

### Patient populations

Participants in the included studies had hypertension (n=13), diabetes mellitus (n=10), obesity or overweight (n=4), hyperlipidaemia (n=3), cerebrovascular disease (n=1), arthritis (n=1), asthma (n=1) or cancer (n=1). Several studies included participants with multiple NCDs (n=5), most commonly combinations of hypertension and diabetes, or diabetes with hyperlipidaemia, with some cases including three or more conditions. See online supplemental table S3 in appendix for more study characteristics details.

### Equity, diversity and inclusion

Equity-related patterns were evident across studies. Engagement with app- or remote support was lower among older adults and rural participants because of low digital literacy and unstable connectivity.^35–37^ Rural settings also faced higher travel and opportunity costs.^15 38 39^ Although VHV or home-visit models helped, they were capacity-limited. Cultural and religious context shaped acceptability: Buddhist-framed approaches were associated with increased motivation in Buddhist-majority areas.^40–42^ Low income, shift work and privacy concerns in cohesive communities reduced attendance.^43 44^ Samples involved often skewed towards women and older adults, leaving male and working age/oldest population under-represented.^39 44^ Several studies excluded low-literacy participants or those with severe comorbidity, limiting equity of benefit.^15 37–39 42 43 45^

EDI factors such as digital literacy, rural–urban location and religious context influenced engagement and intervention outcomes, highlighting the need for culturally and contextually tailored approaches.

### CMOCs: Mechanisms, barriers and facilitators

16 included papers generated 19 CMOCs and grouped them into six cross-cutting programme theories. Table 2 presents the compact PT map of the recurring CMO patterns across settings. (online supplemental table S4 and S5) in the appendix lists study-specific CMOCs and related barriers and facilitators. The following sections present each concept’s results alongside the related barrier and facilitator CMOCs across the included studies. Feedback from two Thai doctoral students (online supplemental table S6) has been reviewed and added to refine some statements, noted as SE-defined (online supplemental table S5) for details).

**Table 2:** CMOCs and PTs with implementation barriers and facilitators

#	Programme theory (PT)	Context (C)	Mechanism (M)	Outcomes (O)	Notes (barriers/facilitators)
1	Self-efficacy and confidence	Cultural-aligned community mindfulness; tailored education and ongoing counselling; visual feedback	Identity-congruent practice and achievable plans build self-belief	Increased literacy/optimism/self-efficacy; reduced BP; weight loss	Barriers: short duration; resource-intensive; low e-literacy and unstable internet (if on app). Facilitators: paced sessions; low-tech or combined with VHV support (SE); Buddhist-congruent framing; literacy-matched materials.
2	Social support and validation	Support from groups; peers/elders; family; staff endorsement	Social proof and belonging normalise new routines	Increased diet or exercise maintenance; cessation support	Barriers: drop-off after groups end; variable family engagement; entrenched norms. Facilitators: regular VHV and family follow-ups (SE); peer or elder leaders; staff endorsement.
3	Motivation	Goals; progress visibility; personalised feedback	Emotional support via success and recognition	Increased commitment and participation	Barriers: over-structured content; repetitive meals; festival periods disrupt routines. Facilitators: pace to readiness (SE); visible progress with feedback; pre-planned culturally acceptable festival strategies (SE).
4	Accountability	Self-monitoring; digital tracking; regular check-ins with staff or VHVs	External or visual prompts reinforce responsibility	Increased adherence to meds, appointments, diet/exercise	Barriers: rural staffing limits intensity; tech unfamiliarity; poor connectivity; no maintenance follow-up. Facilitators: brief remote check-ins by calls or SMS (SE); low-tech blends (SE); low-intensity maintenance check-ins.
5	Emotional resilience	Group-guided mindfulness and meditation; gratitude with cultural fit; Continued support from trusted individuals	Stress regulation plus social connection	Decreased stress and anxiety; increased coping and adherence	Barriers: meditation unfamiliarity; festival-food pressures; short duration; logistics. Facilitators: trusted facilitators; gradual onboarding (SE); ongoing social support; anticipatory festival planning (SE).
6	Relevance and engagement	Cultural and personalised fit; local facilitators involvement; daily routine integration	Acceptability and low burden promote sustained use	Enhanced engagement; improved diet and well-being outcomes	Barriers: time demands; entrenched food norms; religious diversity. Facilitators: cultural tailoring; trusted local facilitators; secular health framing (SE); integration into daily routines; periodic follow-up.

BP, blood pressure; SE, Self efficacy; VHV, village health volunteer.

#### Programme theory 1: self-efficacy through culturally aligned counselling

In contexts where counselling is culturally meaningful, literacy-sensitive, and integrated into everyday routines, individuals develop greater confidence and perceived capability to manage health behaviours, leading to improved adherence, empowerment, and sustained self-management.

Counselling that explicitly builds self-efficacy included culturally grounded mindfulness and positive psychology,^40^ personalised planning^35 45^ or structured self-monitoring with visual feedback.^37 39 46 47^ These interventions improved health literacy, empowerment, and clinical or behavioural outcomes (eg, reduced systolic blood pressure, sustained weight loss, better adherence). Gains were strongest when materials *matched to participants’ literacy levels* and everyday routines. However, short duration and low digital literacy limited long-term persistence. Stakeholder reviewers noted that religion-congruent design, such as practices recognised during Buddhist Len in CMOC 1, strengthens confidence.

#### Programme theory 2: social support through family and community engagement

In contexts where family members, peers, VHVs, and trusted healthcare providers actively participate in care, social validation and collective encouragement reinforce healthy behaviours, leading to greater adherence, wellbeing, and maintenance of lifestyle change.

Group activities, family involvement and trusted community figures encouraged participation and normalised health behaviours.^48^ Peer and elder roles were vital: group programmes fostered shared problem-solving and a sense of belonging, supporting consistent exercise and dietary change,^41^ while locally nominated peers and trained family members increased happiness, self-care and maintenance of healthy habits.^44^ Culturally adapted mindfulness with family participation strengthened family well-being and social trust.^40^ Validation from healthcare staff also mattered: nurse-led interventions improved adherence to smoking cessation.^15^ However, rooted behaviours, variable family engagement and short programme duration reduced long-term impact. Two Thai doctoral students’ feedback emphasised that these effects rely on regular follow-up by VHVs or family. CMOCs further elaborate that intervention effectiveness depends on the frequency and intensity of follow-up.

#### Programme theory 3: motivation through personalised reinforcement and visible progress

In contexts where interventions are personalized, emotionally meaningful, and supported through regular encouragement and visible improvements, individuals experience greater emotional investment and motivation, leading to increased participation and sustained engagement in health behaviours.

Motivation was sustained through personalisation, gratitude-based reflection, visible health improvements, and regular contact (eg, home visits, follow-up calls). Culturally grounded group sessions with gratitude, positive thinking and weekly goal setting fostered emotional investment and sustained commitment,^40^ and redesigning hospital meals for taste, variety and aesthetics improved dietary adherence and health markers.^49^ Regular home visits, whether for lifestyle counselling^43^ or tailored exercise and recreation,^38^ maintained higher participation and physical activity through ongoing encouragement and personalised plans. However, overly structured programmes could be overwhelming, with repetition reducing satisfaction, and motivation often declined once visits ended. Traditions also disrupted diet change and follow-up, as stakeholders pointed to region-specific norms like northern alcohol traditions and southern fishing or food culture. These norms can weaken motivation if not considered.

#### Programme theory 4: accountability through monitoring and continuous follow-up

In contexts where regular monitoring, structured feedback, and follow-up are embedded into daily routines, external prompts reinforce responsibility and behavioural consistency, leading to improved adherence, continuity of care, and sustained behaviour change.

Structured monitoring, regular feedback and visible progress tracking reinforced participants’ responsibility for sustaining health behaviours.^46–48^ Daily tools like calendars, weighing logs and diaries, while paired with feedback from VHVs or trained staff, improved adherence to medication, diet and exercise.^39 43^ Family-monitored diaries and sticker tracking further integrated accountability into everyday routines, reducing smoking and promoting healthier behaviours, though family engagement varied.^15^ Goal setting with staff support helped improve dietary awareness and reduce unhealthy intake, but adherence declined when follow-up ended.^41^ Digital and remote monitoring, such as app-assisted home visits, enhanced medication adherence, blood pressure control, and care continuity,^37^ yet high logistical demands, technical unfamiliarity, and provider training needs limited scalability. Across contexts, the intensity of monitoring was key to success but challenging to sustain long-term. Stakeholder input highlighted overstretched rural staffing (3–5 staff per sub-district) and unreliable internet as capacity issues.

#### Programme theory 5: emotional resilience through trusted and culturally meaningful support

In contexts where emotional support is delivered through trusted relationships and culturally resonant approaches, individuals develop coping strategies, emotional regulation, and social connectedness, leading to improved wellbeing, resilience, and sustained engagement with self-management behaviours.

Mindfulness, meditation and gratitude practices, especially when culturally resonant and delivered by trusted people, fostered emotional regulation, social connection and coping, with reductions in stress and improved adherence. Guided meditation-healing exercises with group encouragement and mindfulness with gratitude practices in Buddhist community settings enhanced mood, family well-being and coping.^40 42^ Continuous emotional and social support from trusted figures, such as community health volunteers during crises or home-based caregivers using digital tools, reduced isolation, improved adherence and stabilised emotional health.^36 37^ Digital self-management programmes plus stress reduction and personalised planning lowered anxiety and improved blood pressure,^35^ while counselling tailored to emotional readiness supported behaviour change and resilience.^45^ However, unfamiliarity with meditation, festival-based food traditions, limited digital literacy, logistical barriers, short programme durations and work demands constrained sustained engagement and broader applicability. Stakeholders confirmed that trusted, culturally aligned delivery is critical to trigger coping and adherence.

#### Programme theory 6: relevance and engagement through contextual relevance and cultural tailoring

In contexts where interventions align with cultural norms, personal readiness, and everyday routines, individuals perceive programmes as more acceptable, relevant, and manageable, leading to increased engagement, satisfaction, and adherence to behaviour change interventions.

While tailoring to cultural norms, personal readiness and routines increased satisfaction, engagement and adherence.^48^ Examples included co-designed meals reflecting patient preferences,^49^ stage-matched counselling for overweight women^45^ and culturally grounded group activities led by trusted facilitators.^40 44^ Other approaches integrated culturally familiar meditation into routine care^42^ or used relatable examples in healthy eating and activity education.^41^ These strategies improved dietary behaviours, lipid control, health literacy, daily functioning and psychological well-being. Embedded dietary norms, competing responsibilities and short implementation periods limited sustained change. Stakeholder reviewers recommended secular health framing in religiously diverse areas to ensure inclusivity, with CMOCs in this concept emphasising the practical implications of this religious-context factor.

#### Barriers and facilitators

Barriers clustered at two levels. Individual-level barriers included low health/eHealth literacy, norms that conflict with intervention goals and limited capacity to engage with intensive programmes. System-level barriers included rural staff shortages and the loss of support after short-term programmes. Facilitators focused on accessibility, cultural alignment and sustainability: simplified formats, cultural tailoring (including religious context), streamlined programme demands, task-sharing with community members, hybrid delivery and planned long-term check-ins.

Four barrier and facilitator CMOCs were added based on two Thai doctoral students (SE-refined in table 2). These CMOCs covered brief context assessments to enable tailored design and engagement; low-tech or combined delivery with VHV support to promote digital components; targeted urban–rural strategies to ease access burdens; and design adaptations that reduce poverty- and time-related costs to improve participation.

## Discussion

This review synthesised 19 CMOCs into six key PTs: self-efficacy and confidence, social support and validation, motivation, accountability, emotional resilience and relevance and engagement within Thailand’s NCD interventions. Across the included studies, these programme theories were commonly associated with practical and family-centred education, routine self-monitoring with feedback, stress-regulation strategies and culturally or personally tailored intervention content. Stakeholder feedback from two Thai doctoral students with professional experience in NCD nursing supported the contextual relevance and feasibility of the PTs. Their feedback highlighted the importance of service capacity, cultural alignment with family, religion and diet and accessibility for people with lower health or digital literacy. They also suggested that intervention benefits may diminish when ongoing support is withdrawn or when delivery exceeds available service capacity.

### Interpretation of emerging PTs

Evidence relating to self-efficacy was drawn from quasi-experimental studies, randomised controlled trials (RCTs) and qualitative research.^35 39 40 46 47^ Digital self-management programmes were associated with improved empowerment among digitally literate urban adults,^35^ while culturally grounded counselling and Buddhist mindfulness may improve confidence in rural family-based settings.^40^ These findings suggest that self-efficacy may operate as an important mechanism for behaviour change, although the contexts that activate this mechanism appear to differ across digital and community-based interventions. This interpretation is supported by previous Thai studies showing that family-oriented diabetes education improved self-efficacy and glycaemic control,^50^ pharmacist-led family interventions promoted treatment adherence^51^ and app-based family support reduced blood pressure.^52^ However, limited digital health literacy among older adults and rural populations may constrain engagement with digitally supported interventions, which potentially limited opportunities for self-monitoring and feedback that underpin self-efficacy.^53^

Social support was consistently found across quasi-experimental and qualitative studies,^40 41 44 48^ especially through peer groups, family roles and trusted facilitators. For example, semi-urban programmes that included peer support were associated with improved well-being and adherence,^44^ while family-integrated mindfulness interventions were associated with social trust.^40^ However, the engagement varied, as some families were less able to sustain long-term involvement, which potentially limited the intervention durability.^41^ This interpretation is supported by wider Thai evidence, showing that family-based interventions improved treatment adherence and HbA1c,^51 52^ although disparities in social support persist among ethnic minority populations and people with renal-complicated hypertension.^54^ Overall, these findings suggest that social support may operate as an important mechanism for behaviour change, although its effectiveness appears to depend on sustained family or VHV involvement.

Motivation appeared across qualitative, quasi-experimental and trial evidence.^38 40 43 49^ Personalised support, including home visits, group sessions and hospital meal redesign, was associated with increased gratitude and treatment adherence, although motivation often declined without continued reinforcement. Similarly, temple-based programmes reported short-term improvements that were less evident without routine follow-up.^51 52^ Cultural factors also emerged to influence motivation. Culturally adapted diabetes prevention programmes were associated with improved adherence in semi-urban Muslim communities,^55^ whereas festivals and regional norms, such as alcohol use at northern festivals and high-salt diets in southern Thailand, may have reduced sustained engagement.^6 56 57^ Based on these findings, it suggested that motivation may work as a context-dependent mechanism supported by ongoing and culturally aligned reinforcement within local community settings.^58^

Accountability was most evident across RCTs, cohort studies^37 39 43^ and several quasi-experimental studies,^46 47^ where diaries, calendars and digital monitoring were associated with sustained adherence through structured feedback. Rural RCTs reported that daily weighing supported by VHVs was associated with greater weight loss,^39^ while digital WinCare networks were associated with improved blood pressure control.^37^ However, resource demands, workforce shortage and limited internet access may restrict the implementation of these approaches, especially in rural subdistricts.^59^ This interpretation is supported by wider evidence, showing that home blood pressure monitoring improved outcomes in urban settings,^60 61^ although national guidelines also recognise challenges related to its standardisation and uptake.^62^ The findings indicate that accountability may work most effectively when structured monitoring and feedback are feasible within local service capacity.

Emotional resilience was supported primarily from quasi-experimental and community-based interventions.^40 42 45^ Buddhist meditation, gratitude practices and mindfulness were associated with improvements in stress, family well-being and coping, particularly when delivered by trusted facilitators.^40 42^ This interpretation is supported by previous evidence, showing that mindfulness reduced blood pressure in pre-hypertensive adults,^63^ while relaxation techniques improved well-being among postmenopausal women.^64^ However, engagement with these approaches may be influenced by digital unfamiliarity, competing work demands and religious diversity. Beyond religion, some Thai studies also suggested that cultural identity may shape emotional resilience. For example, Tai Lue adults with hypertension experienced barriers to self-care related to salt-rich dietary traditions and lower health literacy, which were associated with lower adherence than among non-ethnic populations.^54^ Taken together, these findings suggest that emotional resilience may operate most effectively when interventions are culturally familiar and delivered by trusted facilitators.

Engagement appeared across RCTs and qualitative studies.^41 45 49^ Co-designed dietary interventions were associated with improved satisfaction and adherence,^49^ while stage-tailored counselling contributed to improving dietary outcomes among women.^45^ Qualitative studies further highlighted that culturally grounded group activities were associated with greater engagement,^40 44^ although established dietary norms and competing responsibilities may have limited sustainability.^41^ This interpretation is supported by evidence, showing that food literacy programmes improved HbA1c among older adults,^65^ while culturally tailored interventions for Muslim populations increased the acceptability of diabetes prevention and hypertension management programmes.^48 55^ Mixed-methods research also emphasised the importance of trusted delivery through VHVs, family and familiar settings, while suggesting that workload and continuity gaps may reduce programme effectiveness when interventions are not adapted to local service capacity.^22^ Surveys also identified literacy gaps among ethnic minority groups,^54^ highlighting the importance of collaboration, inclusion and culturally responsive approaches while designing and delivering lifestyle counselling interventions.

### Interpretation of barriers and facilitators

Key barriers identified in this review included low health and digital literacy, cultural norms that sometimes conflicted with intervention goals, programme intensity and capacity constraints, staff shortages and limited post-programme support. These findings were broadly consistent with previous evidence from Thailand. First, lower health and digital literacy may reduce comprehension and self-efficacy, which have been associated with poorer clinical outcomes.^54^ Older adults in middle-income settings, including Thailand, have also been reported to have lower levels of digital health literacy, which may limit engagement with e-health interventions.^53^ Second, cultural practices, such as social drinking and festive customs, may sometimes conflict with therapeutic goals. Survey studies of the national Buddhist Lent alcohol abstinence campaign suggest that behaviour change may be facilitated when interventions are aligned with religious norms, although adherence appears to vary across communities and alcohol use may persist despite religious identification.^56 57 66^

Finally, several health system challenges emerged across the included studies that may influence the sustainability of lifestyle counselling interventions. Longer and more complex programmes helped to place greater demands on participant attendance and implementation fidelity. For example, a cluster randomised controlled trial reported early improvements in risk factors that were not sustained at 24 months after structured support was reduced.^58^ Similarly, although VHVs play an important role in supporting lifestyle counselling, workforce pressures and competing demands within primary care may limit opportunities for ongoing follow-up and reinforcement, contributing to lower effective coverage for diabetes and hypertension.^59 67^ These findings suggest that intervention benefits may diminish once structured support, feedback or monitoring is withdrawn.^58^

Alongside these barriers, the review also identified several facilitators that may support the accessibility, cultural appropriateness and sustainability of lifestyle counselling interventions. Plain-language, practical educational materials supported the improvement of health literacy,^68 69^ while culturally tailored approaches, including religious or family-centred framing, such as Buddhist Lent or family obligations, may promote the relevance of interventions.^56 57^ Similarly, streamlined, lower-intensity programmes with clearly defined goals were associated with reducing implementation burden and improving delivery fidelity.^58^ Task-sharing with VHVs and family members also emerged as an important mechanism for maintaining ongoing support where workforce capacity was limited.^68 69^ Hybrid delivery, combining brief face-to-face contacts with low-tech follow-up and longer-term monitoring, was associated with sustained engagement and maintenance of behaviour change across the included studies.^58 60 64^

### Equality, diversity and inclusive implications

Equity considerations across the included studies centred on digital access and literacy, healthcare accessibility and cultural appropriateness. The findings suggest that improving accessibility through low-tech or hybrid delivery, support from VHVs and approaches that reduce travel and time barriers (such as mobile or satellite clinics and flexible service delivery) may facilitate engagement, particularly for rural populations. Culturally aligned communication, including culturally resonant or secular framing where appropriate, may further improve accessibility among people with lower health literacy and diverse cultural or religious backgrounds. As the included studies predominantly involved women and older adults and frequently excluded individuals with severe comorbidities, the transferability of the PTs may be limited for other populations. Future research could seek broader representation, including men, working-age adults, the oldest age groups and people with more complex health needs.

### Limitations

Although this review developed culturally grounded mechanisms for NCD prevention in Thailand, it has several limitations. First, the rapid nature of the review, while enabling timely theory development, may have resulted in some relevant evidence being missed, particularly unpublished studies or studies not identified through the search strategy. Second, although sixteen studies informed programme theory development, the included studies varied in their intervention characteristics, reported outcomes and representation of different NCDs. Accordingly, the evidence supporting individual programme theories may be varied across mechanisms, with some programme theories being informed by a broader range of evidence than others. In addition, the findings may be more applicable to commonly represented conditions, such as hypertension and diabetes, than to less frequently studied NCDs. Third, relatively few studies reported long-term follow-up, which limited the understanding of how the identified mechanisms operate beyond the intervention period and how programme effects may be sustained over time. Finally, although stakeholder engagement with healthcare professionals helped to assess the contextual relevance and feasibility of the programme theories, patients and members of the public were not involved in the review. Therefore, the perspectives of adults living with NCDs, family caregivers and other community stakeholders were not represented. The programme theories developed through this review should therefore be interpreted in the context of the available evidence and may benefit from further refinement and evaluation in future research.

### Conclusion

This rapid realist review identified six PTs that explain how lifestyle counselling interventions may support behaviour change and self-management among adults living with NCDs in Thailand. The findings suggest that culturally tailored approaches, family and community support, sustained follow-up, hybrid low-tech delivery and the involvement of existing VHVs may help maintain engagement and support behaviour change. These factors may be important across different contexts. Plain-language communication and culturally aligned intervention content also emerged as important mechanisms for improving accessibility, particularly among people with lower health or digital literacy and those living in rural settings. Taken together, these findings provide insight into the contextual factors and mechanisms that may influence the delivery of lifestyle counselling interventions in Thailand. As these PTs are explanatory rather than definitive, they may benefit from further refinement and evaluation in future research.

## Supplementary material

10.1136/bmjopen-2025-115071 online supplemental table 1

10.1136/bmjopen-2025-115071 online supplemental table 2

## Data Availability

All data relevant to the study are included in the article or uploaded as supplementary information.
