# Supplementary material for: How lifestyle counselling supports behaviour change among Thai adults with non-communicable diseases: a rapid realist review
Source: BMJ Open. 2026 Sep 9;16(9):e115071. doi: 10.1136/bmjopen-2025-115071 (PMC13561012; doi:10.1136/bmjopen-2025-115071)
Supplement: Supplementary file 1 [file bmjopen-2025-115071.reviewer_comments.pdf]

PEER REVIEW HISTORY

BMJ Open publishes all reviews undertaken for accepted manuscripts. Reviewers are asked to complete a checklist review form and are provided with free text boxes to elaborate on their assessment. These free text comments are reproduced below.

ARTICLE DETAILS

Title (Provisional)

How Lifestyle Counselling Supports Behaviour Change Among Thai Adults with Non-Communicable Diseases: A Rapid Realist Review

Authors

Chen, Bonan; Angkurawaranon, Chaisiri; Papachristou Nadal, iliatha

---

VERSION 1 - REVIEW

**Reviewer**	**1**
**Name**	**AbdulRaheem, Yousif**
**Affiliation**	**University of Baghdad, Family & Community medicine**
**Date**	**09-Feb-2026**
**COI**	**None**

The topic is timely, important, and highly relevant, and has many valuable implementations. The followings are my main concerns:

1- The manuscript does not adequately explain how “rigour” was assessed, how lower-quality but high-relevance studies were handled, or how potential bias influenced CMOC development. No risk-of-bias assessment or equivalent analytic judgement is reported.

2- Initial screening relied largely on a single reviewer, with partial double-checking. In combination with an English-only search, this raises concerns about missed or misclassified studies, particularly in a country-specific review.

3- Only 13 studies were included over a 20-year period, which is a concern for the comprehensiveness of the search and the achievement of theoretical saturation.

4- The low inclusion rate (13 out of 7,268 studies screened) and the lack of clarity in the exclusion criteria affect the transparency of the inclusion process.

5- The exclusion of doctoral theses and studies that lack outcome data do not align with the realist approach to methodology, which favors a variety of evidence that is process rich.

6- The exclusion of studies due to “unreliable evidence” is not operationally defined

7- Many studies had 4-8 week follow-up only.

8- The link between individual studies and specific CMOCs is not clearly presented

9- Missing quantitative data - sample sizes, effect sizes, study quality ratings absent

10- Tables are overly dense and, in some cases, difficult to interpret.

**Reviewer**	**2**
**Name**	**Hews-Girard, Julia**
**Affiliation**	**University of Calgary, Faculty of Nursing**
**Date**	**07-Apr-2026**
**COI**	**None**

Thank you for the opportunity to review this manuscript detailing a rapid realist review examining how lifestyle counselling supports behaviour change.

I have several questions/comments for the author's consideration.

1) Realist work, including reviews, are theory driven. This paper is currently lacking this key component of realist synthesis.

2) I'm not sure that two doctoral students represent 'the public' in this case. Can the authors provide more information as to why these individuals were chosen/asked to participate and why they appropriately represent the public (adults with non-communicable disease).

3) please provide the RAMSES checklist

4) CMOCs are provided in IF... then... because... formats (as the authors have done). Addition in the "however" is unnecessary given the authors' inclusion of CMOCs for the barriers and negative outcomes. Consider reframing these.

5) There are multiple references missing from the methods, including references to the seminal work on how to conduct and report realist reviews. There are also references missing from the tables of the CMOCs indicating which of the included articles contributed to each CMOC.

6) Please specify how rigour/quality of the articles was assessed and provide references for the tool used to do the assessment.

VERSION 1 - AUTHOR RESPONSE

Reviewer 1<br>Dr. Yousif AbdulRaheem, University of Baghdad			
Reviewer Comment	Response / How Addressed	Action Taken	Revised in Manuscript (Page & Line Numbers)

1- The manuscript does not adequately explain how "rigour" was assessed, how lower-quality but high-relevance studies were handled, or how potential bias influenced CMOC development. No risk-of-bias assessment or equivalent analytic judgement is reported.	We sincerely thank the reviewer for raising this important methodological point! We appreciate the opportunity to clarify the approach used in this realist review. Unlike conventional systematic reviews, realist review methodology does not typically rely on formal risk of bias checklists or hierarchical appraisal tools. Instead, evidence is assessed according to its relevance, rigour, credibility, and explanatory contribution to the development and refinement of CMOCs and programme theories, consistent with realist review principles (Pawson et al., 2005; Saul et al., 2013).	The manuscript was revised to further clarify the realist approach to evidence appraisal, including the assessment of relevance and rigour in relation to programme theory development. Reference to the RAMESES guidance has also been added to strengthen methodological transparency and reporting rigour.	Page 8/Line 191-200; Page 10/Line 210-212; Table 1 in supplementary material; Table 3 on Page 9
2- Initial screening relied largely on a single reviewer, with partial double-checking. In combination with an English-only search, this raises concerns about missed or misclassified studies, particularly in a country-specific review.	We thank the reviewer for raising this important point! We would like to clarify that study screening was conducted by both BC and IPN, including during the original dissertation phase of the project. We recognise that the methods section did not describe this process clearly enough, which may have given the impression that screening relied primarily on a single reviewer. We have therefore revised the manuscript to clarify the roles of both reviewers in the screening and eligibility assessment process.<br><br>We also acknowledge that restricting inclusion to English-language	The manuscript has been revised to (1) include Thai-language studies within the eligibility criteria, (2) clarify the screening and assessment procedures, including the involvement of both BC and IPN throughout the screening process, and (3) update the PRISMA flow diagram, final study count, and study characteristics table accordingly.	Page 2/ Line 38; Page 7/Line 169-173, 177-178; Page 8/ Line 188-190; Table 3 on Page 9; Page 12/Line 262-266; Page 13/Line 294; Page 22/Line 494-495; Figure 1 in the supplementary material; Table 4 in the appendix

	studies may have excluded relevant country-specific evidence. As part of the revision process, we revisited Thai-language records identified during the original search and re-screened them against the predefined inclusion criteria with support from a Thai-speaking reviewer. This additional review resulted in the inclusion of three further Thai-language studies that met the eligibility criteria. These studies were consistent with and further supported the existing programme theories identified in the review.		
3- Only 13 studies were included over a 20-year period, which is a concern for the comprehensiveness of the search and the achievement of theoretical saturation.	We thank the reviewer for this observation. We acknowledge the concern regarding the relatively small number of included studies and the implications for search comprehensiveness and theoretical saturation. The review employed a systematic search across multiple databases, supplemented by citation searching and screening of Thai-language papers. Following the reviewer's suggestion, we revisited the Thai-language records identified during the original search process and included additional eligible studies. Despite only 16 studies met the inclusion criteria, it reflects the relatively limited published literature on lifestyle counselling and	The manuscript was revised to improve transparency of the search and selection process, including Thai-language studies, updates to the PRISMA flow diagram and final study count, and clearer explanation of programme theory refinement and theoretical saturation within the Methods and Results section.	Page 2/ Line 38; Page 7/Line 169-173; Page 8/ Line 188-190; Table 3 on Page 9; Page 12/Line 262-266; Page 10/Line 222-231; Page 13-18/Line 294-295, 302, 304-307, 316-319, 331-336, 349-352, 366-371, 386-390; Page 22/Line 494-495; Figure 1 in the supplementary material; Table 4 in the appendix

	health coaching interventions for NCDs in Thailand rather than a restricted search strategy.<br><br>Regarding the realist review methodology, the aim was theory development and refinement rather than exhaustive aggregation of outcomes (Pawson et al., 2005; Saul et al., 2013; Wong et al., 2013). The included studies generated 19 context–mechanism–outcome configurations (CMOCs) and six programme theories, which provide sufficient explanatory depth to address the review objectives. Furthermore, the additional studies identified during the revision process largely reinforced existing mechanisms and contextual explanations rather than generating substantially new programme theories, suggesting that an adequate level of theoretical saturation had been achieved.		
4- The low inclusion rate (13 out of 7,268 studies screened) and the lack of clarity in the exclusion criteria affect the transparency of the inclusion process.	Thank you for this important observation. We agree that greater transparency regarding the inclusion and exclusion process would strengthen the clarity and reproducibility of the review. In response, we revised the eligibility criteria and screening description to provide a more explicit account of the study selection process. For example, the original table only	Specifically, we expanded the intervention criteria to explicitly define eligible behavioural and lifestyle-related interventions, added realist review-specific criteria relating to programme theory and CMOC development, and introduced methodological	Page 2/ Line 38; Page 7/Line 169-173; Page 8/ Line 188-190; Table 3 on Page 9; Page 12/Line 262-266; Page 13/Line 294; Page 22/Line 494-495; Figure 1 in the supplementary material; Table 4 in the appendix

	specified inclusion based on population, intervention, study design, language, and publication date. The revised criteria additionally assessed whether studies provided contextual, mechanistic, or outcome-related evidence relevant to CMOC development and programme theory refinement, and whether they contained sufficiently rigorous methodological and explanatory detail to support realist synthesis.<br><br>We also incorporated the country-specific evidence and therefore included both English- and Thai-language studies within the review process, with Thai-language papers screened and assessed with support from the Thai co-author to ensure contextual relevance and accuracy.	rigour criteria to study selection.<br><br>We also include both English- and Thai-language papers and clarified the screening and assessment procedures for Thai-language studies. In addition, the PRISMA flow diagram, final study count, and study characteristics table have been updated to reflect the inclusion of the additional Thai-language studies.	
5- The exclusion of doctoral theses and studies that lack outcome data do not align with the realist approach to methodology, which favors a variety of evidence that is process rich.	We sincerely thank the reviewer for this thoughtful observation. We acknowledge that realist reviews can integrate diverse forms of evidence, including grey literature, where relevant to programme theory development. However, in this review, we prioritised peer-reviewed empirical studies to ensure methodological robustness, credibility, and sufficient contextual and explanatory detail to support reliable CMOC and programme theory	The manuscript has been revised to clarify the rationale for prioritizing peer-reviewed empirical evidence and to better explain how relevance, rigour, and explanatory contribution were considered in relation to CMOC development and programme theory refinement.	Table 3 on Page 9

	development. Consistent with realist review principles, evidence was selected based on its relevance and rigour in contributing to explanatory understanding, rather than publication type alone (Pawson et al., 2005; Wong et al., 2013). This approach is also reflected in more recent realist review protocols and applications, which emphasize the importance of contextually rich and methodologically transparent evidence for theory refinement (Elfghi et al., 2026).		
6- The exclusion of studies due to “unreliable evidence” is not operationally defined.	Thank you for this important observation. We agree that further clarification regarding the definition of “unreliable evidence” would improve the transparency of the review process.<br><br>In response, we added a paragraph to clarify the criteria used when assessing study reliability and determining exclusion decisions. We now explain that studies were considered unreliable when there were substantial concerns regarding methodological integrity, transparency, or credibility of findings, including insufficient methodological description, inconsistent reporting, unsupported conclusions, or inadequate linkage between findings and	As a result, we added a new paragraph in the Methods section to clarify the operational definition of “unreliable evidence” and the criteria used for exclusion decisions, including considerations of methodological integrity, transparency, credibility, and relevance to programme theory refinement.	Page 8/ Line 191-200

	outcomes. We also clarified that, consistent with realist review principles, studies were not excluded solely based on study design hierarchy, but rather on the trustworthiness and relevance of the evidence for programme theory development and refinement.		
7- Many studies had 4-8 week follow-up only.	We sincerely thank the reviewer for pointing out this important issue! We had also identified this limitation within the manuscript. We all agree that shorter follow-up periods may limit the ability to fully assess sustained behaviour change, particularly for individuals living with NCDs, where the development of self-management skills, self-efficacy, and long-term lifestyle adaptation often requires extended support and observation over time.	The limitation regarding short follow-up duration and its implications for long-term behaviour change and self-management outcomes has been emphasized within the Limitations section.	Page 25/Line 549-551, 555-556
8- The link between individual studies and specific CMOCs is not clearly presented.	Thank you for highlighting this important issue. We agree that clearer linkage between individual studies and specific CMOCs is essential to improve transparency and allow readers to better understand how mechanisms and programme theories were developed from the included evidence. In response, we revised the presentation of the CMOC tables to make the contribution of individual studies more explicit and transparent.	We have added references throughout the specific CMOC tables and alongside each CMOC to clearly identify the studies informing each mechanism and programme theory. Additional citations and explanatory detail were also integrated into the Results section to improve transparency of the analytic process and interpretation of mechanisms.	Table 6 and 7 on Page 5-10 in the appendix

9- Missing quantitative data - sample sizes, effect sizes, study quality ratings absent.	We sincerely thank the reviewer for this valuable observation. We acknowledge that additional study-specifc detail would improve the transparency and readability of the review, and we appreciate the importance of demonstrating rigour in evidence appraisal. However, within rapid realist review (RRR) methodology, the primary purpose of included studies is not quantitative comparison or formal quality ranking alone, but the extraction and interpretation of contexts, mechanisms, and outcomes (CMOCs) that contribute to programme theory development.<br><br>Consistent with realist review principles, studies are therefore assessed according to their relevance, credibility, and explanatory contribution to understanding how and why interventions work across different contexts, rather than through conventional methodological hierarchies or risk-of-bias tools alone (Pawson et al., 2005; Saul et al., 2013). Nevertheless, we attempted to again ensure transparency and methodological rigour, and therefore revised the presentation of study characteristics and included additional reporting guidance to support clearer	We have revised Table 4 (Study Characteristics) to improve transparency and readability. We added information on participant numbers and study design for each included study, alongside a more concise presentation of population, intervention, comparison, and outcome characteristics.<br><br>The manuscript was revised to further clarify the realist approach to evidence appraisal and CMOC development. In addition, a completed RAMESES reporting checklist has now been included as supplementary material to strengthen methodological transparency and reporting rigour.	Table 4 on Page 2-4 in the appendix; Table 1 in supplementary material; Table 3 on Page 9

	interpretation of the included evidence.		
10- Tables are overly dense and, in some cases, difficult to interpret.	Thank you for these helpful suggestions. We agree that Table 4 was overly descriptive and may have limited the clarity and accessibility of key study information. In response, we revised the table to improve conciseness and readability.	We eventually revised Table 4. The study characteristics table has been substantially condensed and reorganized to present clearer and more concise information on population, intervention, comparison, and outcomes across included studies.	Table 4, Page 2-4 in the appendix

Reviewer 2.<br>Dr. Julia Hews-Girard, University of Calgary			
Reviewer Comment	Response / How Addressed	Action Taken	Revised in Manuscript (Page & Line Numbers)
1) Realist work, including reviews, are theory driven. This paper is currently lacking this key component of realist synthesis.	We thank the reviewer for this insightful comment. We agree that the initial programme theory section required stronger theoretical grounding and clearer linkage to established behavioural and implementation theories. We also recognized the importance of framing the findings as programme theories rather than presenting mechanisms as purely thematic concepts. In response, we strengthened the theoretical framing of the manuscript by linking key mechanisms, such as self-efficacy, motivation, and	The manuscript was revised to include a clearer Initial Programme Theories subsection, strengthen links to established behavioural theories and behaviour change frameworks, clarify the iterative process of programme theory refinement in the Methods section, and reframe the Results around programme theories rather than thematic concepts.	Page 5-6/Line 109-131; Page 10/Line 223-224; Page 13-18/Line 294-295, 302, 304-307, 316-319, 331-336, 349-352, 366-371, 386-390

	accountability, to relevant substantive theories and behaviour change frameworks. We also clarified the realist review process by describing how initial programme theories were iteratively tested and refined using CMOCs, contradictory findings, and stakeholder feedback.		
2) I'm not sure that two doctoral students represent 'the public' in this case. Can the authors provide more information as to why these individuals were chosen/asked to participate and why they appropriately represent the public (adults with non-communicable disease).	Thank you for this important observation. We recognize that the previous wording may have unintentionally implied that these contributors represented the wider public or adults living with non-communicable diseases (NCDs). We have revised the manuscript to clarify their role more accurately.<br><br>The two contributors were Thai doctoral students with professional backgrounds as diabetes and NCD nurses working within the Thai healthcare system. Both had direct clinical experience supporting adults living with NCDs and were invited to contribute contextual and implementation insights relevant to healthcare delivery and lifestyle interventions in Thailand. Their involvement was therefore intended to provide professional and contextual expertise rather than to represent the broader patient	We have amended the manuscript to clarify the rationale for their inclusion and have acknowledged this distinction within the limitations section. All terms of "Public involvement" or "PI" have been changed to "Stakeholder engagement" and "SE".	Page 2/Line 46-48; Page 3/Line 69; Page 11-12/Line 248-257; Page 19-20/Line 422-424; Page 25-26/ Line 551-555, 570-572, 578, 595; Table 6, Table 7, Table 8 in the appendix

	population or public perspectives.		
3) please provide the RAMSES checklist.	Thank you for noticing this important point! We agree that including a realist review reporting checklist is essential to ensure transparency, completeness, and methodological rigour in realist reviews and evaluations. We also recognize that adherence to recognized reporting standards is particularly important for BMJ publication standards.	We adapted and completed a realist review RAMESES II checklist based on Wong et al. (2013) and Simionato et al. (2023). The completed checklist has now been included as supplementary material.	Table 1 in supplementary material
4) CMOCs are provided in IF... then... because... formats (as the authors have done). Addition in the "however" is unnecessary given the authors' inclusion of CMOCs for the barriers and negative outcomes. Consider reframing these.	We thank the reviewer for pointing this out. We acknowledge that the use of "HOWEVER" statements within CMOC tables reflects a newer writing style sometimes used in rapid realist review research to demonstrate contrasting contextual findings. However, we also recognize that this format has not yet been widely adopted or formally standardized in published realist review reporting. We agree that including these statements both within the tables and again in the Results section may have resulted in unnecessary repetition and reduced readability.	In response, all "HOWEVER" statements were removed from Table 6 and the specific CMOC tables to improve clarity and readability. The explanatory and contrasting findings are now presented only within the relevant programme theory sections of the Results, where they are discussed in greater analytical detail.	Table 6 on Page 5-8 in the appendix
5) There are multiple references missing from the methods, including references to the seminal work on how to conduct and report realist reviews. There are also references missing from the tables of the	We sincerely thank the reviewer for this very important observation. We agree that clearer linkage between individual studies, CMOCs, and programme theories would strengthen the transparency and	Citations have now been added throughout all CMOC tables to clarify the connections between individual studies and each CMOC. More detailed programme theory analysis, with supporting citations,	Page 6-7/Line 150-152, 154-157; Table 6 and 7 on Page 5-10 in the appendix

CMOCs indicating which of the included articles contributed to each CMOC.	interpretability of the review. In response, we have added further references related to rapid realist review methodology within the Methods section and clarified how evidence from included studies informed CMOC and programme theory development.	has also been merged into the Results section.	
6) Please specify how rigour/quality of the articles was assessed and provide references for the tool used to do the assessment.	We sincerely thank the reviewer for highlighting this important methodological point. In this rapid realist review, article rigour was assessed according to realist review principles, focusing on the relevance, credibility, and explanatory contribution of each study to the development and refinement of CMOCs and programme theories, rather than through a conventional risk-of-bias checklist alone (Pawson et al., 2005; Saul et al., 2013). Studies were assessed based on whether the evidence provided sufficiently trustworthy and meaningful data to support programme theory development. To strengthen transparency and reporting rigour, we also applied recognized realist review reporting guidance.	The Methods section has been revised to clarify the approach used to assess rigour and quality in this review, including references to Pawson et al. (2005), Saul et al. (2013), and the RAMESES reporting checklist has also been included as supplementary material for showing rigour.	Page 8/Line 184-188, 191-200; Table 1 in supplementary material; Table 3 on Page 9

VERSION 2 - REVIEW

**Reviewer**	**1**
**Name**	**AbdulRaheem, Yousif**

**Affiliation**	**University of Baghdad, Family & Community medicine**
**Date**	**26-Jun-2026**
**COI**	

The manuscript addresses an important public health topic, but several issues reduce its scientific strength. The evidence base is relatively small, with only 16 studies included, limiting the robustness of the proposed programme theories. In addition, the reported Patient and Public Involvement does not accurately reflect genuine patient or public participation, as the contributors were healthcare professionals. The manuscript also requires substantial language editing to improve clarity and readability. Finally, the review does not indicate the level of confidence or strength of evidence supporting each context–mechanism–outcome configuration, making it difficult for readers to judge which findings are most reliable for informing practice and policy.

**Reviewer**	**2**
**Name**	**Hews-Girard, Julia**
**Affiliation**	**University of Calgary, Faculty of Nursing**
**Date**	**25-Jun-2026**
**COI**	

Thank you for your responses. I have no further comments.

VERSION 2 - AUTHOR RESPONSE

Reviewer 1<br>Dr. Yousif AbdulRaheem, University of Baghdad		
Reviewer Comment	Response / How Addressed	Revised in Manuscript (Page & Line Numbers)
The manuscript addresses an important public health topic, but several issues reduce its scientific strength.<br><br>1- The evidence base is relatively small, with only 16 studies included, limiting the robustness of the proposed programme theories.	1- Thank you for this helpful suggestion. We have revised the manuscript to acknowledge this limitation more explicitly. The Limitations section now states that although 16 studies informed programme theory development, the evidence underpinning	p3, Line 69-72/ p26, Lines 581–588, 590–596; p27, Lines 597–606

2- In addition, the reported Patient and Public Involvement does not accurately reflect genuine patient or public participation, as the contributors were healthcare professionals.	individual context–mechanism–outcome configurations (CMOCs) and programme theories varied across mechanisms and intervention settings. Consistent with the aims of a rapid realist review, the proposed programme theories are intended to provide explanatory insights rather than definitive conclusions. We also state that the programme theories require further refinement and empirical evaluation in future research. In addition, we have revised the Discussion and Conclusion to ensure that our interpretations remain appropriately aligned with the available evidence.	p11–12, Lines 231–265
3- The manuscript also requires substantial language editing to improve clarity and readability.	2. Thank you for this important comment. We agree that stakeholder engagement with healthcare professionals should be distinguished from formal Patient and Public Involvement (PPI). We have therefore revised the manuscript in accordance with *BMJ Open* guidance. A PPI statement has been added to the end of the Methods section stating that no patients or members of the public were involved in this rapid realist review. We have also revised the manuscript throughout to distinguish stakeholder engagement with healthcare professionals from formal PPI.	Throughout manuscript<br><br>p8, Lines 183–203; p10, Lines 207–223; p26, Lines 581–596; Supplementary Tables S4–S5
4- Finally, the review does not indicate the level of confidence or strength of evidence supporting each context–mechanism–outcome configuration, making it difficult for readers to judge which findings are most reliable for informing practice and policy.	3- Many thanks for this feedback. The manuscript has undergone comprehensive language editing to improve clarity, grammar, readability and consistency throughout.<br><br>4- Thank you for this valuable comment. We agree that transparency regarding the evidence supporting each context–mechanism–outcome configuration (CMOC) is important. As recommended for realist reviews, we assessed the relevance and rigour of the studies included to inform programme theory development rather than	

		undertaking a formal certainty-of-evidence assessment. To improve transparency, the supplementary tables identify the studies contributing to each CMOC and programme theory, enabling readers to judge the breadth of evidence underpinning each explanation. We have also clarified in the Limitations section that the evidence supporting individual programme theories varied across the included studies, with some being informed by a broader range of evidence than others. Accordingly, the proposed programme theories should be interpreted as explanatory rather than definitive and warrant further refinement and empirical evaluation.	
