# Supplementary material for: How lifestyle counselling supports behaviour change among Thai adults with non-communicable diseases: a rapid realist review
Source: BMJ Open. 2026 Sep 9;16(9):e115071. doi: 10.1136/bmjopen-2025-115071 (PMC13561012; doi:10.1136/bmjopen-2025-115071)
Supplement: Supplementary file 2 [file bmjopen-16-9-s001.docx]

**RAMESES II checklist for reporting a realist synthesis**

Supplementary Table 1. Summary of realist synthesis reporting standards adapted from Wong et al. and Simionato et al.

| **TITLE** | | | Reported in document  Y/N/Unclear | Page  number |
| --- | --- | --- | --- | --- |
|  |  | Clearly indicate in the title that the study is a realist evaluation. | Y | Page 1 |
| **ABSTRACT** | | | | |
|  |  | While acknowledging publication requirements and house style, this section should briefly outline the policy, programme, or initiative being evaluated, the setting in which it operates, the purpose of the evaluation, the main evaluation questions or objectives, the evaluation approach, methods of data collection and analysis, and the key findings and conclusions.  Where appropriate and required by journals, it may also include brief information about participants, recruitment procedures, and sampling strategies.  The summary or abstract should clearly indicate that a realist evaluation approach was applied and that programme theory was developed, tested, and/or refined throughout the study. | Y | Page 1-3 |
| **INTRODUCTION** | | | | |
|  | Rationale for evaluation | Describe the rationale for conducting the review and explain how it is expected to enhance or add to the current knowledge and understanding of the topic. | Y | Page 3-5 |
|  | Programme theory | Describe the initial programme theory or theories underlying the intervention, policy, or initiative. | Y | Page 5-6 |
|  | Evaluation questions, objectives and focus | State the evaluation questions and objectives and explain how programme theory shaped the scope and focus of the study. | Y | Page 6 |
|  | Ethical approval | Clarify whether ethical approval was required and obtained, including relevant details. If not required, explain the reason. | Y | Page 12 |
| **METHODS** | | | | |
|  | Rationale for using realist evaluation | Describe the reasons for selecting a realist evaluation approach and explain any adaptations made to the methodology, where applicable. | Y | Page 6 |
|  | Scoping the literature | Explain and justify the initial exploratory scoping process used to examine the literature. | Y | Page 7 |
|  | Searching processes | Taking into account the requirements of the target journal or publication, describe and justify the process used for iterative searching. Provide details of all information sources used in the review. For electronic database searches, include information such as the database names, search terms, coverage dates, and the date the searches were last conducted. If experts or individuals with knowledge of the literature or topic area were consulted, explain how they were identified and chosen. | Y | Page 7, Table 2 in the appendix |
|  | Selection and appraisal of documents | Describe the process used to determine which data from documents were included or excluded in the review, and provide a clear justification for these decisions. | Y | Page 7-9 |
|  | Data extraction | Describe and justify the types of data or information extracted from the included documents, explaining why these were selected for the review. | Y | Page 10 |
|  | Data analysis and synthesis processes | Provide a detailed explanation of the analysis and synthesis procedures, including the key constructs examined and the steps involved in the analytical process. | Y | Page 10-11 |
| **RESULTS** | | | | |
|  | Document flow diagram | Provide details on the number of documents assessed for eligibility and included in the review with reasons for exclusion at each stage as well as an indication of their source of origin (for example, from searching databases, reference lists and so on). You may consider using the example templates (which are likely to need modification to suit the data) that are provided. | Y | Figure 1 as a supplementary file |
|  | Document characteristics | Provide information on the characteristics of the documents included in the review. | Y | Table 4 in the appendix |
|  | Main findings | Present the key findings with a specific focus on theory building and testing. | Y | Page 13-19 |
| **DISCUSSION** | | | | |
|  | Summary of findings | Summarise the key findings in relation to the evaluation questions, programme theory, purpose, and intended audience. | Y | Page 19-20 |
|  | Strengths, limitations and future research directions | Discuss both the strengths and weaknesses of the review. This should include, but is not limited to, consideration of each stage of the review process and an assessment of the overall quality and robustness of the evidence underpinning the explanatory findings. Any limitations identified should also highlight areas where additional research or further investigation may be required. | Y | Page 3, 24-25, |
|  | Comparison with existing literature | Compare and contrast the findings with previous studies or literature on similar programmes or interventions. | Y | Page 20-23 |
|  | Conclusion and recommendations | List the main implications of the findings and place these in the context of other relevant literature. If appropriate, offer recommendations for policy and practice. | Y | Page 25 |
|  | Funding and conflict of interest | Provide details of funding, the role of funders, and any potential conflicts of interest among the evaluators. | Y | Page 26 |
