# Supplementary material for: How lifestyle counselling supports behaviour change among Thai adults with non-communicable diseases: a rapid realist review
Source: BMJ Open. 2026 Sep 9;16(9):e115071. doi: 10.1136/bmjopen-2025-115071 (PMC13561012; doi:10.1136/bmjopen-2025-115071)
Supplement: Supplementary file 3 [file bmjopen-16-9-s002.docx]

**Appendices**

**Table S2. Full Search Strategy for Electronic Databases**

| **Database** | **Search Strategy** | **Limits/**  **Filters Applied** | **Search Date(s)** | **Records Retrieved (n)** |
| --- | --- | --- | --- | --- |
| **Scopus** | TITLE-ABS-KEY (("non-communicable disease*" OR NCD OR "chronic disease*" OR "cardiovascular disease*" OR "heart disease" OR hypertension OR diabetes OR obesity OR "respiratory disease*" OR asthma OR cancer OR "chronic kidney disease*") AND ("lifestyle intervention*" OR "lifestyle counselling" OR "lifestyle counselling intervention*" OR "health coaching" OR "health counselling") AND (Thailand OR Thai) AND (adult* OR "older adult*" OR "young adult*")) | Publication years 2005–2025; English language | 16–19 January 2025; updated June 2025 | 6000 |
| **Google Scholar** | ("lifestyle intervention*" OR "lifestyle counselling" OR "lifestyle counselling intervention*" OR "health coaching" OR "health counselling") AND (Thailand OR Thai) AND (diabetes OR hypertension OR cardiovascular disease OR NCD OR "chronic disease") | Publication years 2005–2025 | 16–19 January 2025; updated June 2025 | 1247 |
| **ProQuest** | (("non-communicable disease*" OR NCD OR "chronic disease*" OR diabetes OR hypertension OR obesity OR cardiovascular disease) AND ("lifestyle intervention*" OR "lifestyle counselling" OR "lifestyle counselling intervention*" OR "health coaching" OR "health counselling") AND (Thailand OR Thai) AND (adult*)) | Publication years 2005–2025; English language | 16–19 January 2025; updated June 2025 | 229 |
| **PubMed** | (("non-communicable diseases" OR NCD OR diabetes OR hypertension OR obesity OR cardiovascular disease OR chronic disease) AND ("lifestyle intervention*" OR "lifestyle counselling" OR "lifestyle counselling intervention*" OR "health coaching" OR "health counselling") AND (Thailand OR Thai)) | Humans; publication years 2005–2025; English language | 16–19 January 2025; updated June 2025 | 119 |
| **EMBASE (Ovid)** | (('non communicable disease'/exp OR diabetes/exp OR hypertension/exp OR obesity/exp OR cardiovascular disease/exp) AND ('lifestyle intervention*' OR 'lifestyle counselling' OR 'lifestyle counselling intervention*' OR 'health coaching' OR 'health counselling') AND (Thailand OR Thai)) | Humans; publication years 2005–2025; English language | 16–19 January 2025; updated June 2025 | 27 |
| **ThaiJO** | Thai-language equivalents of non-communicable diseases, lifestyle interventions, lifestyle counselling, lifestyle counselling interventions, health coaching, health counselling, diabetes, hypertension, cardiovascular disease, and Thailand | Publication years 2005–2025 | 16–19 January 2025; updated June 2025 | 16 |

**Additional exclusion criteria:** Systematic reviews, review articles, and case studies were excluded during database searching where database functionality permitted and were otherwise removed during title and abstract screening.

**Note:** Initial searches were conducted between 16 and 19 January 2025. An updated search was conducted in June 2025 to identify any newly published eligible studies prior to final manuscript submission. However, the June 2025 update search did not identify any additional studies meeting the inclusion criteria.

**Table S3. Summarytable of Sixteen Included Studies**

| **Study (Author, Year)** | **Study Design** | **Population (P)** | **Intervention (I)** | **Comparison (C)** | **Outcomes (O)** |
| --- | --- | --- | --- | --- | --- |
| **Intarakamhang & Macaskill (2021)** | Cluster randomized control trials (RCTs) | Adults 41–59 with obesity, hypertension (HT), or diabetes (n=200) | Transformative health literacy + mindfulness + behaviour modification + positive psychology + family activities | Standard education | Improved health literacy (HL), behaviours, psychological capital (hope, optimism, and resilience), blood pressure (BP), glucose |
| **Prajaknate & Kachentawa (2022)** | Mixed-methods survey | Adults 31-40 with HT, hyperlipidemia, diabetes mellitus (DM), cardiovascular disease (CVD) (n=180+5 key informants) | eHealth literacy + CHV home visits + online consultations + social support | None (survey-based) | Better self-efficacy and self-management |
| **Chotisiri et al. (2022)** | Experimental study | Older adults 58–72 with HT, DM, overweight, arthritis(n=45) | Home visits + diet education + tailored exercise + recreational activities | Routine care | Better exercise and self-care; limited dietary changes |
| **Rodjanathum & Krasae-In (2024)** | Pilot study | Patients with various NCDs (HT, DM, hyperlipidaemia, CVD, multimorbidity) (number not reported) | Low-GI meal redesign + sensory food improvement ± supervised exercise | Two active arms | Improved blood lipids, BP, and glucose; exercise outperforms diet-only. |
| **Jayasvasti et al. (2021)** | Qualitative study | Adults 50–65 with pre-HT or pre-DM (n=46) | Group lifestyle education + goal-setting + self-monitoring + peer/family involvement + staff coaching | None (qualitative) | Better health behaviours with improved BP/glucose; struggled to sustain changes after follow-up. |
| **Chotisiri et al. (2016)** | Randomized control trials (RCTs) | Older adults 50–80 with HT (n=69) | Laughter therapy/relaxation + mild exercise + dietary self-monitoring + home visits | Routine care | Improved cholesterol, HDL and LDL; limited BP changes. |
| **Artsanthia & Sari (2019)** | Quasi-experimental study | Elderly with DM or HT (n=196) | Meditation-healing practice (SKT) + monthly follow-up + reminders + peer encouragement | Pre–post only (no parallel control) | Reduced stress, BP, glucose |
| **Sakboonyarat et al. (2022)** | Prospective cohort study | Adults ≥60 with HT or type 2 diabetes (T2D) (n=98) | WinCare home visits + BP/weight monitoring + education + reminders + app support | Usual care | Better BP control, medication adherence and quality of life; BMI did not differ significantly. |
| **Liampeng et al. (2023)** | Randomized control trials (RCTs) | Adults 18–60 with obesity (n=107) | Self-weighing with calendar feedback + weekly VHV counselling + goal-setting | Usual care | Reduced body weight and BMI; Limited differences in waist indices and BP |
| **Polsook & Aungsuroch (2024)** | Randomized control trials (RCTs) | Adults 25-90 with HT (n=48) | Mobile self-management app + tailored education + action plans + scheduled follow-up calls | Standard care | Reduced systolic BP and anxiety, stronger self-management confidence; limited diastolic BP differences |
| **Karintrakul & Angkatavanich (2017)** | Randomized control trials (RCTs) | Women 19-60 with overweight or obesity (n=50) | Individualised nutrition counselling matched to a transtheoretical model (TTM), + self-monitoring tools + phone follow-ups | Handbook only | Better weight management and reduced body fat with less energy or macronutrient intake. |
| **Silawan et al. (2024)** | Quasi-experimental study | Older adults 60-93 with HT, DM, hyperlipidaemia or other CVD (n=229) | Peer-led wellness groups (trained supportive peers, self-help groups, family training, community activities). | None | Better wellbeing, Activities of Daily Living (ADL), healthier diet, and physical activity; limited depression and alcohol use |
| **Aung et al. (2019)** | Randomized control trials (RCTs) | Adults 35–80 smokers with DM, HT or both (n=319) | Nurse-led motivational counselling + CO (Smokerlyzer) feedback + family-assisted diary monitoring + optional nicotine replacement therapy (NRT). | Routine care | Higher smoking quit rate; minimal attrition indicates good acceptability. |
| **Lahpunsa et al. (2024)** | Quasi-experimental study | Muslim patients with uncontrolled HT (n=56) | Self-management + family + VHV participation | Routine care | Improved health behaviours and BP |
| **Phulthong et al. (2023)** | Quasi-experimental study | CKD stage 3 with DM/HT (n=40) | Self-management + home visits | Pre-post only | Improved self-management and self-efficacy |
| **Treekham et al. (2021)** | Quasi-experimental study | Difficult-to-control diabetes (n=15) | Self-management support programme | Pre-post only | Improved behaviour and HbA1c |

**Table S4. Study-specific CMOCs on lifestyle counselling interventions**

| **No.** | **Programme theories (PTs)** | **CMOC #** | **Statements** |
| --- | --- | --- | --- |
| PT 1 | Self-Efficacy and Confidence (N=7) | CMOC 1  [42] | **IF** adults (with low health literacy in rural Thailand) participate in mindfulness-based and positive psychology interventions grounded in Buddhist tradition,  **THEN** they show increased health literacy, optimism, self-efficacy, and improved physiological measures (e.g., fasting blood sugar, systolic BP),  **BECAUSE** *identity-congruent practice builds psychological capital and moral commitment (SE-defined)***.** |
|  |  | CMOC 2  [37, 48, 49] | **IF** adults *with adequate digital literacy and stable connectivity (SE-defined)* receive digital personalised planning and self-management training (app),  **THEN** they report greater empowerment, reduced anxiety, and lower systolic blood pressure,  **BECAUSE** tailored planning reinforces confidence and self-efficacy. |
|  |  | CMOC 3  [39, 41, 47] | **IF** individuals with overweight or obesity engage in self-monitoring routines (e.g., daily weight tracking, dietary reflection) supported by structured education and *regular (weekly/fortnightly) counselling contacts (SE-defined)*, **THEN** they are more likely to demonstrate weight loss, improved dietary habits, and sustained adherence to lifestyle changes, **BECAUSE** the combination of personalised guidance, visual feedback, and behavioural reinforcement increases confidence and motivation for self-management. |
| PT 2 | Social Support and Validation (n=5) | CMOC 4  [43, 46, 50] | **IF** older adults participate in group-based education supported by peers and family *plus periodic VHV/family follow-up (SE-defined),*  **THEN** they engage more consistently in exercise and dietary changes,  **BECAUSE** peer and family encouragement normalize new routines. |
|  |  | CMOC 5  [15] | **IF** patients receive *regular (SE-defined)* nurse-led motivation, family-monitored diaries, and feedback monitoring,  **THEN** they achieve better maintenance of new routines such as smoking cessation,  **BECAUSE** family support and visual feedback foster accountability and social validation. |
|  |  | CMOC 6  [42, 46] | **IF** older adults receive *ongoing (SE-defined)* support from locally nominated peers and trained family members,  **THEN** they report higher happiness, better self-care, and sustained healthy behaviours,  **BECAUSE** trusted peer involvement and cultural fit build validation. |
| PT 3 | Motivation (n=4) | CMOC 7  [42] | **IF** gratitude, positive thinking, and weekly goal setting are embedded in culturally grounded group and *paced to participants’ readiness (SE-defined),*  **THEN** participants are more emotionally invested and motivated in behaviours change,  **BECAUSE** reinforcement and reflective practice sustain commitment. |
|  |  | CMOC 8  [51] | **IF** food-related intervention is redesigned for taste, variety, and aesthetics based on patient feedback,  **THEN** dietary adherence and health markers improve,  **BECAUSE** motivation increases when meals are satisfying and personalized. |
|  |  | CMOC 9  [40, 45] | **IF** older adults receive weekly home visits with tailored exercise and recreation a*nd plans anticipate major festivals with culturally acceptable diet alternatives (SE-defined)*,  **THEN** they maintain greater participation and physical activity,  **BECAUSE** frequent encouragement and personalized plans sustain motivation. |
|  |  |  |  |
| PT 4 | Accountability (n=8) | CMOC 10  [41, 45, 48-50] | **IF** participants use daily checking calendars and receive consistent feedback from VHVs or professional health staff,  **THEN** they maintain improved self-management skills like medication, weight loss, exercise,  **BECAUSE** external and visual accountability reinforce daily behaviours. |
|  |  | CMOC 11  [39, 45] | **IF** participants receive *regular (SE-defined)* digital/remote regular home visits from trained community-based providers to track health indicators, deliver reminders, and provide visual feedback,  **THEN** participants are more likely to adhere to medication, attend appointments, and demonstrate improvements in clinical outcomes such as blood pressure,  **BECAUSE** the combination of consistent face-to-face interaction and digital monitoring enhances accountability, supports behaviour tracking, and reinforces continuity of care across home and health service settings. |
|  |  | CMOC 12  [15] | **IF** a family member monitors using stickers and diaries *with brief caregiver coaching (SE-defined),*  **THEN** participants reduce behaviours such as smoking and sustain changes,  **BECAUSE** accountability is woven into everyday family interactions. |
|  |  | CMOC 13  [41, 43, 48] | **IF** participants set dietary goals and track behaviours with ongoing staff support,  **THEN** they improve dietary awareness and reduce unhealthy intake,  **BECAUSE** structured self-monitoring increases responsibility. |
| PT 5 | Emotional Resilience (n=6) | CMOC 14  [44] | **IF** adults engage in guided meditation-healing exercises with group encouragement *delivered by trusted facilitators (SE-defined)*,  **THEN** they report reduced stress, improved mood, and better physiological indicators,  **BECAUSE** emotional-regulation techniques are practised in a supportive context. |
|  |  | CMOC 15  [42] | **IF** mindfulness and gratitude practices are integrated into Buddhist community interventions,  **THEN** participants show stronger family wellbeing and stress coping,  **BECAUSE** shared emotional tools align with spiritual-cultural values. |
|  |  | CMOC 16  [37-39] | **IF** participants managing chronic health conditions receive consistent support from trusted individuals (e.g., CHVs, homecare providers, counsellors, or app-based follow-up) that fosters relational trust, encouragement, and personalised problem-solving,  **THEN** they report reduced stress and anxiety, improved confidence, and better adherence to self-management behaviours,  **BECAUSE** feeling supported, emotionally validated, and socially connected enhances psychological resilience and motivation. |
| PT 6 | Relevance and Engagement (n=7) | CMOC 17  [43, 47, 50, 51] | **IF** interventions are culturally or personally tailored (e.g., co-designed meals, or stage-matched counselling),  **THEN** participants are more likely to engage, improve dietary behaviours, and achieve behaviour change outcomes (e.g., weight loss, lipid control),  **BECAUSE** personalised, culturally familiar content enhances satisfaction, motivation, and relevance. |
|  |  | CMOC 18  [42, 46, 50] | **IF** interventions are delivered by trusted local facilitators or nominated community members (e.g., supportive peers, health workers aligned with cultural norms),  **THEN** engagement, emotional trust, and confidence improve, leading to greater health literacy and behavioural follow-through,  **BECAUSE** familiar and respected figures increase acceptability and help embed change within community norms. |
|  |  | CMOC 19  [43, 44] | **IF** health interventions are designed to fit smoothly into participants’ daily routines and care patterns (e.g., meditation integrated into routine care),  **THEN** practice adherence, stress reduction, and psychological wellbeing improve,  **BECAUSE** seamless integration reduces burden and reinforces sustainability of new behaviours. |

**Table S5. CMOCs of specific barriers and corresponded facilitators; four SE defined CMOCs**

| **Barriers** | **CMOC #** | **Statements** |
| --- | --- | --- |
| Low Health or Digital Literacy | CMOC 1  [37, 39, 42-44] | **IF** participants with limited health or digital literacy are offered interventions using complex materials or unfamiliar platforms,  **THEN** they may struggle to fully understand or apply health behaviours,  **BECAUSE** low baseline comprehension impedes engagement.  **HOWEVER,** simple language, visual tools, and hands-on demonstrations can improve clarity and confidence, leading to better participation and understanding. |
| Cultural Norms Conflicting with Intervention Goals | CMOC 2  [15, 40, 42, 43, 46] | **IF** intervention goals (e.g., healthy diet, smoking cessation) conflict with entrenched cultural or social norms,  **THEN** participants may resist or disengage from behaviour change efforts,  **BECAUSE** behaviours seen as routine or identity-linked are difficult to alter.  **HOWEVER**, cultural tailoring and integration with religious or community settings increase trust and relevance, encouraging uptake and long-term behaviour change. |
| Overly Intensive or Resource-Heavy Interventions | CMOC 3  [37, 39, 41, 42, 46] | **IF** a lifestyle intervention is multi-phased, highly structured, and requires significant resources (e.g., 10-session programs, extensive facilitator training, large time investment),  **THEN** participants and facilitators may find it difficult to sustain participation and isolate which parts are effective,  **BECAUSE** the information load can overwhelm users and the program may not be feasible in resource-limited real-world settings.  **HOWEVER,** simplifying content, reducing burden, and prioritizing essential components with plans can improve feasibility and uptake. |
| Staff/Resource Shortages (especially in rural areas) | CMOC 4  [38, 39, 41, 45, 46] | **IF** programs rely heavily on professional staff or centralized services,  **THEN** coverage and continuity may falter in rural or under-resourced areas,  **BECAUSE** workforce shortages and travel barriers hinder delivery.  **HOWEVER,** task-sharing with community volunteers or peer educators extends reach, reduces workload, and fosters local ownership. |
| Digital Health/E-Health for Older Adults | CMOC 5  [37-39] | **IF** a self-management program is delivered via a mobile app and relies on participants to use digital tools,  **THEN** older adults or those with low digital literacy may have trouble fully engaging or sustaining self-care behaviours,  **BECAUSE** unfamiliarity with technology, limited health literacy, and a short intervention period pose challenges to uptake and retention.  **HOWEVER,** using simplified interfaces, visual guidance, and hybrid support models can improve engagement and usability. |
|  | CMOC 6  [37-39] | **IF** remote support (e.g., online consultations, eHealth literacy training) is provided to older adults with NCDs,  **THEN** some may struggle to access or trust online health information,  **BECAUSE** baseline eHealth literacy is low and participants have little experience with technology, especially during abrupt transitions such as COVID-19 lockdowns.  **HOWEVER,** combined approaches that combine digital and face-to-face support can build trust and bridge digital skill gaps. |
| Lack of Ongoing Support or Short Duration | CMOC 7  [15, 43, 45] | **IF** participants receive group-based education and support only during the short intervention period (with little or no follow-up after the program ends),  **THEN** many struggle to maintain behaviour changes over time,  **BECAUSE** the loss of ongoing encouragement, accountability, and practical support leads to decreased motivation and a return to previous habits.  **HOWEVER,** regular check-ins, peer engagement, and family reinforcement can extend the impact and encourage sustained behaviour change. |
|  | CMOC 8  [37, 40, 43, 45] | **IF** intensive support such as weekly home visits is limited to a short-term program with minimal aftercare,  **THEN** behaviour change may not be sustained after the intervention ends,  **BECAUSE** participants face barriers and relapses when ongoing reinforcement and reminders are withdrawn.  **HOWEVER,** integrating long-term check-ins such as text messages, booster sessions, or caregiver involvement can support continuity and relapse prevention. |
| SE-refined* | CMOC 9 | **IF** implementers conduct a brief context assessment (e.g., local norms, resources, connectivity, workforce),  **THEN** the intervention is tailored and engagement improves,  **BECAUSE** design aligns with cultural practices and delivery constraints. |
|  | CMOC 10 | **IF** participants with low digital literacy or unstable internet receive low-tech or combined delivery with VHV scaffolding (including paper tools, phone calls, brief in-person coaching),  **THEN** anxiety drops and adherence rises,  **BECAUSE** scaffolding substitutes for missing digital skills and maintains accountability. |
|  | CMOC 11 | **IF** programmes use targeted urban–rural strategies (e.g., satellite or mobile sessions; timing aligned to rural work patterns),  **THEN** attendance and continuity improve,  **BECAUSE** travel and time costs fall. |
|  | CMOC 12 | **IF** programme design reduces out-of-pocket and time costs (e.g., free or low-cost access; short, well-timed sessions),  **THEN** participation and retention improve,  **BECAUSE** opportunity-cost barriers are lowered. |

**Table S6.** Stakeholder feedback from two thai doctoral students with matched questions

| **Questions for Stakeholders** | **Feedback Overview** | |
| --- | --- | --- |
| Q1: Overall, what do you think of the concepts we have identified? From a Thai perspective, are these concepts familiar or relevant to you, and why? Was there anything that surprised you? | PT 1: Self-efficacy and confidence | Validated: Buddhist-based mindfulness and positive psychology can enhance optimism and self-management skills, especially in rural areas with low health literacy. |
|  |  | Barriers: Low digital literacy among older adults; limited access to and familiarity with complex web platforms. |
|  |  | Recommendations:  Use practical, culturally relevant examples (e.g., alcohol abstinence during Buddhist Lent, meat avoidance on observance days).  Prefer low-tech mobile apps over web-based platforms for inclusivity. |
|  | PT 2: Social support and validation | Validated: Family co-living and peer encouragement align with Thai collectivist culture to sustain behaviour change. |
|  |  | Barriers: Peer supporters with lived experience are rare in the Thai health system. |
|  |  | Recommendations:  Increase peer support capacity in future programmes.  Strengthen nurse-led interventions in rural areas; address ingrained habits like smoking and alcohol use with targeted motivational strategies. |
|  | PT 3: Motivation | Validated: Gratitude practices, goal setting, and home visits are valuable for encouraging behaviour change. |
|  |  | Barriers: Weekly home visits may be unrealistic due to village health volunteer workload constraints. |
|  |  | Recommendations:  Balance intervention intensity with feasibility.  Explore alternative low-burden strategies for ongoing motivation. |
|  | PT 4: Accountability | Validated: Positive reinforcement and supportive feedback are valued in sustaining engagement. |
|  |  | Barriers: Cultural norms may make overt encouragement less common in Thai communication styles. |
|  |  | Recommendations:  Provide staff training in culturally appropriate supportive communication techniques. |
|  | PT 5: Emotional resilience | Validated: Buddhist mindfulness and meditation can support emotional regulation and mental health. |
|  |  | Barriers: Trust in temples may be lower in some communities; approaches need to be sensitive to religious diversity (e.g., Muslim-majority areas). |
|  |  | Recommendations:  Emphasise therapeutic/mental health benefits over religious framing.  Provide sustained resources and guidance for regular practice. |
|  | PT 6: Relevance and engagement | Validated: Personalised, culturally tailored intervention plans improve engagement and outcomes. |
|  |  | Barriers: Regional, religious, and rural–urban differences in traditions, infrastructure, and resources. |
|  |  | Recommendations:  Conduct preliminary context assessments before implementation.  Adapt delivery to local infrastructure and cultural norms. |
| Q2: Do you think these concepts are widely used and effective in Thailand? | Validated: NCD prevention interventions are already widely used in Thailand. | |
|  | Effectiveness factors:  Varies across contexts, influenced by traditions, lifestyle, urban–rural location, and socio-economic status.  Poverty can limit participation due to affordability and access barriers. | |
|  | Rural-specific challenges:  Limited or unreliable internet reduces the feasibility of telemedicine and digital care.  Heavy reliance on family members, village health volunteers (VHVs), and village heads for delivery.  These local actors often have competing responsibilities, reducing consistency of care. | |
|  | Recommendations:  Conduct preliminary context assessments before implementation to ensure fit with local traditions, resources, and infrastructure.  Ensure delegation of responsibilities is proportionate and supported with adequate training and resources. | |
| Q3: Are there significant variations in how these interventions work (for example, between rural and urban areas, or among different regions, traditions, lifestyles, or habits)? | Rural vs. Urban | Rural areas rely on lay VHVs with limited training (3–5 per sub-district) and overstretched public health staff. Most care is delegated to local actors, who only escalate serious cases to professionals. |
|  |  | Urban areas benefit from better healthcare access, multiple insurance schemes, and larger hospitals with specialised staff. |
|  | Regional differences | Distinct traditions and habits influence health behaviours, such as alcohol norms in the North, fishing culture in the South, and fast-paced lifestyles in Bangkok. |
|  | Religious diversity | Buddhist-based interventions may need to be reframed in secular terms for Muslim-majority areas. |
|  | Age preferences | Older adults often prefer traditional or religious approaches and may resist technology-based interventions. Lower digital literacy in older populations can make tech-heavy programmes burdensome. |
| Q4: Are you aware of any additional relevant studies or empirical papers from the past 20 years in Thailand that you think we should consider including? | No. | |
